# Supplementary material for: Ecklonia stolonifera Okamura Extract Suppresses Myocardial Infarction-Induced Left Ventricular Systolic Dysfunction by Inhibiting p300-HAT Activity
Source: Nutrients. 2022 Jan 28;14(3):580. doi: 10.3390/nu14030580 (PMC8838613; doi:10.3390/nu14030580)
Supplement: Supplementary file 1 [file nutrients-14-00580-s001.zip › nutrients-1541795-supplementary.pdf]

Supplemental Table S1. Primers using for Quantitative RT-PCR

primer	base sequence
rat-ANF-Fw	ATCACCAAGGGCTTCTTCCT
rat-ANF-Rv	CCTCATCTTCTACCGGCATC
rat-BNP-Fw	TTCCGGATCCAGGAGAGACTT
rat-BNP-Rv	CCTAAAACAACCTCAGCCCGT
rat-α-SMA-Fw	AGCTCTGGTGTGTGACAATG
rat-α-SMA-Rv	TTTGGCCCATTCCAACAATC
rat-Col 1A1-Fw	CCAGTTCGAGTATGGAAGCGA
rat-Col 1A1-Rv	GTAGGCTACGCTGTTCTTGCA
rat-HPRT1-Fw	GATTTTATCAGACTGAAGAGCTACTG
rat-HPRT1-Rv	TCCAACAAAGTCTGGCCTGT
